# Visit-to-visit HbA_1c_ variability is associated with in-stent restenosis in patients with type 2 diabetes after percutaneous coronary intervention

**DOI:** 10.1186/s12933-020-01111-7

**Published:** 2020-09-04

**Authors:** Chen Die Yang, Ying Shen, Lin Lu, Zhen Kun Yang, Jian Hu, Rui Yan Zhang, Wei Feng Shen, Feng Hua Ding, Xiao Qun Wang

**Affiliations:** 1 Department of Cardiology, Ruijin Hospital, Shanghai Jiao-Tong University School of Medicine, 197 Ruijin Road II, Shanghai, 200025 People’s Republic of China; 2 Institute of Cardiovascular Disease, Shanghai Jiao-Tong University School of Medicine, Shanghai, People’s Republic of China

**Keywords:** HbA_1c_ variability, In-stent restenosis, Type 2 diabetes, Percutaneous coronary intervention, Diameter stenosis

## Abstract

**Background:**

Patients with type 2 diabetes are under substantially higher risk of in-stent restenosis (ISR) after coronary stent implantation. We sought to investigate whether visit-to-visit HbA_1c_ variability is a potential predictor of ISR in diabetic patients after stent implantation.

**Methods:**

We consecutively enrolled type 2 diabetic patients who underwent successful elective percutaneous coronary intervention and performed follow-up coronary angiography after around 12 months. The incidence of ISR and its relationship with visit-to-visit HbA_1c_ variability, expressed as coefficient of variation (CV), standard deviation (SD) and variability independent of the mean (VIM), were studied. Multivariable Cox proportional hazards models were constructed to analyze the predictive value of HbA_1c_ variability for ISR.

**Results:**

From September 2014 to July 2018 in Ruijin Hospital, a total of 420 diabetic patients (688 lesions) after stent implantation were included in the final analysis. During a mean follow-up of 12.8 ± 1.3 months, the incidence of ISR was 8.6%, which was significantly increased in patients with higher CV of HbA_1c_ (*P *= 0.001). The mean diameter stenosis (DS), net luminal loss and net luminal gain were 22.9 ± 16.8%, 0.42 ± 0.88 mm and 1.66 ± 0.83 mm, respectively. Greater DS was observed in subjects with higher tertiles of CV of HbA_1c_ (*P *< 0.001), and this trend was more prominent in patients with optimal glycemic control (HbA_1c_ ≤ 7%) in the baseline. In multivariate analysis, HbA_1c_ variability was independently associated with incidence of ISR after adjustment for traditional risk factors and mean HbA_1c_ (HR: 3.00 [95% CI 1.14–7.92] for highest vs. lowest tertile). Inclusion of CV of HbA_1c_ led to a better risk stratification accuracy. Assessing HbA_1c_ variability by SD or VIM yielded similar findings.

**Conclusions:**

This study suggests that visit-to-visit HbA_1c_ variability is an independent predictor of incidence of ISR in patients with type 2 diabetes after stent implantation.

*Trial registration* NCT02089360: NCT

## Background

Patients with type 2 diabetes are under substantially increased risk of rapid-progressive and diffuse atherosclerosis [1, 2], myocardial infarction [3] and poor coronary collateralization [4]. After percutaneous coronary intervention (PCI) and deployment of stents, diabetic patients are predisposed to exaggerated neointimal hyperplasia and the development of in-stent restenosis (ISR) [5, 6]. In the era of drug-eluting stents (DES), although restenosis rate has significantly declined, diabetic patients still suffer from higher risk of ISR than non-diabetic patients [7, 8]. The prognosis of diabetic patients after DES implantation is also more dismal than that of non-diabetic patients, with increased rates of cardiac death, myocardial infarction, target lesion failure and target vessel revascularization [9].

Hyperglycemia is a critical contributory factor to the development of restenosis [10], partly attributed to endothelial dysfunction [11], excessive production of reactive oxygen species [12] and formation of advanced glycation end-production [13]. Pre-procedural optimal glycemic control was shown to be associated with lower rate of stent failure in comparison with suboptimal control patients [10]. A retrospective study analyzing glycemic control based on sequential HbA_1c_ measurements from preprocedural to 6-month follow-up also suggested that sustained glycemic control is associated with better clinical outcomes in diabetic patients after PCI [14].

On the other hand, emerging evidence suggests that glycemic variability confers an additional risk to diabetic complications, which is predicted by mean glucose levels alone and may, to some extent, underlie the pathogenesis of micro- and macro-vascular diabetic complications. In the short-term, glycemic variability assessed by continuous glucose monitoring or serial glucose levels during hospitalization is associated with poor prognosis in patients with coronary artery disease (CAD) [15–18]. In the long-term, a retrospective study analyzing data from Diabetes Control and Complications Trial (DCCT) demonstrated that HbA_1c_ variability adds to mean HbA_1c_ in predicting the development of retinopathy and nephropathy in type 1 diabetes [19]. A prospective study of cohort of type 2 diabetes from Renal Insufficiency and Cardiovascular Events (RIACE) revealed that HbA_1c_ variability affects chronic kidney disease more than average HbA_1c_ [20]. Recent studies further showed that long-term glycemic variability, either estimated by serial measurements of fasting plasma glucose or by HbA_1c_, is a strong predictor of all-cause mortality and cardiovascular events [21–23]. However, the relationship between glycemic variability and ISR is still unclear. Therefore, in the present study, we sought to investigate whether visit-to-visit HbA_1c_ variability is a potential predictor of ISR in patients with type 2 diabetes after DES implantation.

## Methods

### Study population

A total of 920 consecutive patients with type 2 diabetes and CAD were screened, who received follow-up coronary angiography ~ 12 months after DES-based PCI of de novo lesions in native coronary arteries between September 2014 and July 2018 from the database of Advanced Glycation Endproducts and Development of CAD Program (AGENDA) in Ruijin Hospital, Shanghai. Patients were referred to coronary angiography for the evaluation of established or suspected CAD due to typical chest pain, positive exercise stress test, or positive myocardial perfusion scan. ISR was defined as recurrence of luminal diameter stenosis (DS) of > 50% within the stent or in the 5-mm proximal or distal segments adjacent to the stent at follow-up angiography.

**Fig. 1:**
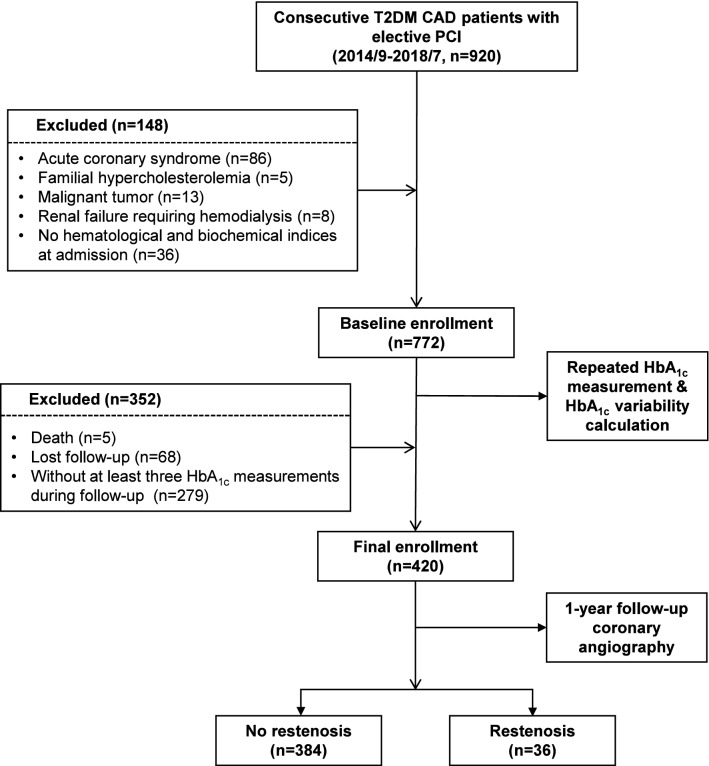
Flow chart of recruitment procedure. T2DM, type 2 diabetes mellitus; CAD, coronary artery disease; PCI, percutaneous coronary intervention; HbA_1c_, glycated hemoglobin A_1c_

For the purpose of this study and to avoid confounding serum data, patients who had acute coronary syndrome (n = 86) during initial angiography and PCI, familial hypercholesterolemia (n = 5), malignant tumor (n = 13), or renal failure requiring hemodialysis (n = 8) were excluded. Another 36 subjects with no hematological and biochemical indices at admission were further excluded. All these patients received a quarterly clinical evaluation, routine analyses and HbA_1c_ measurements. Follow-up coronary angiography was performed after around 12 months and all the enrolled patients were reminded by telephone in advance. During follow-up, 5 patients died and 68 patients were lost to follow-up. For calculation of HbA_1c_ variability, subjects (n = 279) without at least three HbA_1c_ measurements during follow-up (≥ 3 months apart) were also excluded. The remaining 420 subjects constituted the study population (Fig. 1). The diagnosis of type 2 diabetes was made according to the criteria of American Diabetes Association [symptoms of diabetes with casual plasma glucose concentration ≥ 200 mg/dL (11.1 mmol/L) or fasting plasma glucose ≥ 126 mg/dL (7.0 mmol/L), 2 h postprandial glucose ≥ 200 mg/dL (11.1 mmol/L) during an oral glucose tolerance test, and currently or previously treated with insulin and/or oral hypoglycemic agents] [24]. Hypertension was diagnosed according to seventh report of the Joint National Committee on prevention, detection, evaluation, and treatment of high blood pressure (JNC 7).

This study complies with the Declaration of Helsinki. The study protocol was approved by the local hospital ethics committee, and written informed consent was obtained from all participants.

### Baseline clinical and biochemical assessments

Blood samples were obtained at the day of angiography in all patients after an overnight fasting and collected in a quiet, air-conditioned room after at least 20 min supine rest. Serum glucose, insulin, blood urea nitrogen, creatinine, uric acid, total cholesterol, low-density lipoprotein cholesterol (LDL-C), high-density lipoprotein cholesterol (HDL-C), triglycerides, apolipoprotein A-I and apolipoprotein B were assessed (HITACHI 912 Analyzer, Roche Diagnostics, Germany). The estimated glomerular filtration rate (eGFR) was computed using the Chronic Kidney Disease Epidemiology Collaboration equation [25]. Blood HbA1c concentration was measured using ion-exchange high performance liquid chromatography with Bio-rad Variant Hemoglobin Testing System (Bio-Rad Laboratories, USA). Serum N-terminal pro-B-type natriuretic peptide (NT-proBNP) was determined using a commercially available electrochemiluminescence immunoassay kit (Roche Diagnostics). Serum levels of high sensitive C-reactive protein (hsCRP) were determined by ELISA (Biocheck Laboratories, Toledo, OH, USA). The detailed information about medical history and lifestyles including smoking status was obtained using a standard questionnaire by trained physicians. Body mass index (BMI) was calculated using the formula of weight/height^2^ (kilograms per square meter) [26]. Blood pressure was measured on the non-dominant arm in a seated position after a 10-min rest, using an electronic blood pressure monitor (OMRON Model HEM-752 FUZZY’ Omron Co., Dalian, China). Three measurements were taken at 1-min intervals, and the average was used for analysis.

### Angiographic analysis

Coronary angiography was performed using standard Judkins technique through radial or femoral approach. For each patient, multiple matched angiographic views were obtained after intracoronary administration of nitrate. Quantitative coronary analysis (QCA) of all angiographic data before and after procedure and during follow-up was performed (TERRA, GE, USA) by two experienced interventional cardiologists (FH Ding and XQ Wang), who were unaware of clinical information of the patients. Using the outer diameter of the contrast-filled catheter as the calibration, the minimal lumen diameter (MLD) and reference diameter (RD) in diastole before intervention was determined from multiple projections by interpolated method. Lesion length was measured as the distance (in millimeters) from the proximal to distal shoulder in the projection with the least amount of foreshortening. The lesion was stented using a normal-to-normal technique, usually including 5-mm-long, angiographically normal segments proximal and distal to the lesion. Net luminal loss was defined as the difference between the MLD immediately after the procedure and that measured during follow-up. Net luminal gain was defined as the difference between the MLD before the procedure and that measured during follow-up. A value of 0 mm was assigned for MLD in the case of total occlusion at baseline. For patients who underwent multi-lesion coronary angioplasty, the most severe restenotic lesion at follow-up was entered into the analysis.

### HbA_1c_ variability determinations

HbA_1c_ was measured in the baseline and during follow-up period for at least three times in 3-month interval. Then the mean and variability of HbA_1c_ were calculated. Three measures of HbA_1c_ variability were employed for the analysis. Intraindividual variability of HbA_1c_ was primarily defined as intraindividual coefficient of variation (CV) of HbA_1c_ across visits. The alternative variability of HbA_1c_ includes: 1) standard deviation (SD) and 2) the variability independent of the mean (VIM), which is calculated by the equation as previously reported [27]: VIM = 100 × SD/mean^β^, where β is the regression coefficient based on natural logarithm of SD on natural logarithm of mean of the study population.

### Statistical analysis

Continuous variables were presented as median (interquartile range) or mean ± SD, and categorical data were summarized as frequencies (percentages). Normal distribution of continuous variables was evaluated by Shapiro–Wilk test. For normally distributed variables, differences in tertiles of HbA_1c_ variability and subgroup analysis were performed by one-way or two-way analysis of variance (ANOVA) followed by post hoc *t* test with Bonferroni correction. For non-normally distributed continuous variables, differences were analyzed by Mann–Whitney U test or Kruskal–Wallis test. Differences in categorical variables were analyzed by χ^2^ test. The association between measures of HbA_1c_ variability and the incidence of ISR was assessed by Cox regression from which hazard ratios (HR) and 95% confidence interval (CI) were calculated. The assumption of proportionality of the Cox model covariates was tested by plotting Schoenfeld residuals. Five models were constructed for each measure of HbA_1c_ variability and binary angiographic restenosis (DS ≥ 50%) was employed as the dependent variable. In model 1, sex and age were adjusted. In model 2, we further adjusted admission systolic and diastolic blood pressure, BMI, non-HDL-C and eGFR. In model 3, additional adjustment was performed with the post-PCI RD of target vessel, total stented length and medication use including oral hypoglycemic agent (OHA) and insulin. In model 4 and 5, we further adjusted for baseline HbA_1c_ and the mean HbA_1c_ level during follow-up, respectively. Net reclassification improvements (NRI) and integrated discrimination improvements (IDI) were analyzed to assess the improvement in clinical utility of the prediction model by considering HbA_1c_ variability. All statistical analyses were performed using the R statistical package v.3.6.3 (R Project for Statistical Computing, Vienna, Austria). A 2-tailed < 0.05 was considered statistically significant.

## Results

### Baseline characteristics of the study population

A total of 420 subjects with 688 lesions, with a mean follow-up period of 12.8 ± 1.3 months, were included in the analysis. The male-to-female ratio was 74:26 and the mean age was 64.5 ± 9.0 years. Among these subjects, 73.8% were with hypertension and 77.6% of the subjects were with multivessel disease. The mean HbA_1c_ during follow-up was 7.4 ± 1.2%, and CV, SD, VIM of HbA_1c_ during follow-up were 0.061 (IQR 0.038–0.107), 0.402 (IQR 0.252–0.839) and 0.209 (IQR 0.127–0.297), respectively. CV (Pearson’s r = 0.325, *P *< 0.001) and SD (Pearson’s r = 0.445, *P *< 0.001) were correlated to the mean HbA_1c_ while there was no significant correlation between VIM and the mean HbA_1c_ level (Pearson’s r = 0.070, *P *= 0.169). To analyze the effect of HbA_1c_ variability on ISR, we divided the population based on tertiles of CV of HbA_1c_ (Table 1). There was no significant difference in age, sex, history of hypertension, admission blood pressure, smoking status and duration of diabetes between the three tertiles. At admission, subjects with the highest tertile of CV of HbA_1c_ had higher levels of HbA_1c_, fasting and 2 h postprandial glucose, but lower 2 h postparandial insulin level than those with the lowest tertile. Fasting insulin level was similar between the three groups. Meanwhile, HDL-C was lower, whereas serum creatine and hsCRP were higher in subjects with the highest tertile. OHA and insulin were more frequently used in subjects with higher CV of HbA_1c_.

**Table 1:** Baseline characteristics

Tertiles of CV of HbA_1c_	T1 (0.005–0.045)	T2 (0.045–0.086)	T3 (0.086–0.397)	*P*
n	141	139	140	
Demographic characteristics and clinical measures				
Male sex	102 (72.3)	110 (79.1)	97 (69.3)	0.161
Age, years	64.79 ± 8.80	63.99 ± 8.87	64.74 ± 9.33	0.705
BMI, kg/m^2^	25.61 ± 3.47	25.14 ± 2.83	25.59 ± 3.14	0.375
Systolic BP, mmHg	139.34 ± 20.09	137.43 ± 19.68	137.24 ± 23.39	0.657
Diastolic BP, mmHg	78.42 ± 13.57	75.96 ± 11.71	75.41 ± 11.18	0.091
Medical history				
Hypertension	101 (71.6)	105 (75.5)	104 (74.3)	0.749
Duration of diabetes, years	11.8 ± 9.8	8.9 ± 5.7	11.1 ± 8.0	0.078
Current smoker	59 (41.8)	76 (54.7)	60 (42.9)	0.058
Laboratory values				
HbA_1C_, %	7.0 ± 1.6	7.1 ± 1.0	8.4 ± 1.5	< 0.001
Fasting glucose, mmol/L	6.97 ± 2.61	6.85 ± 2.31	9.17 ± 3.86	< 0.001
Postparandial glucose (2 h), mmol/L	12.05 ± 3.87	12.57 ± 4.41	14.98 ± 5.04	< 0.001
Fasting insulin, µU/mL	11.03 (8.26–16.99)	9.97 (6.66–16.05)	11.04 (7.39–18.85)	0.451
Postparandial insulin (2 h), µU/mL	46.53 (35.88–75.78)	44.50 (26.59–83.72)	37.61 (24.18–63.65)	0.009
HOMA-IR	3.28 (2.18–5.57)	3.18 (1.73–5.16)	4.62 (2.29–6.68)	0.005
Hemoglobin, g/L	133.99 ± 16.10	131.82 ± 17.46	131.66 ± 20.73	0.489
Triglyceride, mmol/L	1.50 (1.15–2.35)	1.28 (0.98–2.04)	1.70 (1.15–2.13)	0.008
Total cholesterol, mmol/L	4.20 ± 1.17	3.96 ± 1.08	4.10 ± 1.27	0.243
HDL cholesterol, mmol/L	1.06 ± 0.24	1.08 ± 0.28	0.98 ± 0.20	0.002
LDL cholesterol, mmol/L	2.44 ± 0.92	2.30 ± 0.88	2.46 ± 0.93	0.295
Non-HDL cholesterol, mmol/L	3.14 ± 1.16	2.88 ± 1.04	3.11 ± 1.24	0.119
Alanine aminotransferase, IU/L	26.97 ± 16.97	27.03 ± 17.47	28.76 ± 20.16	0.647
Serum creatinine, μmol/L	80.57 ± 18.76	87.34 ± 40.13	103.68 ± 100.37	0.008
Blood urea nitrogen, mmol/L	5.69 ± 1.74	5.66 ± 2.37	6.29 ± 3.05	0.052
eGFR, mL/min/1.73 m^2^	81.80 ± 16.73	82.19 ± 17.54	80.66 ± 20.46	0.775
hsCRP, mg/L	1.21 (0.55–4.24)	1.19 (0.42–4.09)	1.96 (0.91–8.55)	0.009
Cardiac function				
LVEF, %	63.3 ± 8.6	62.6 ± 8.8	62.2 ± 9.7	0.619
Medication use				
Aspirin	136 (96.5)	129 (92.8)	132 (94.3)	0.402
P2Y_12_ inhibitor	129 (91.5)	129 (92.8)	128 (91.4)	0.893
Beta blocker	113 (80.1)	108 (77.7)	100 (71.4)	0.207
ACEI	49 (34.8)	42 (30.2)	56 (40.0)	0.230
ARB	47 (33.3)	65 (46.8)	57 (40.7)	0.072
CCB	45 (31.9)	53 (38.1)	39 (27.9)	0.183
Statin	136 (96.5)	133 (95.7)	136 (97.1)	0.806
OHA	66 (46.8)	57 (41.0)	79 (56.4)	0.034
Biguanides	30 (21.3)	30 (21.6)	42 (30.0)	0.155
Sulfonylureas	22 (15.6)	28 (20.1)	36 (25.7)	0.109
Meglitinides	4 (2.8)	6 (4.3)	8 (5.7)	0.492
Thiazolidinediones	2 (1.4)	2 (1.4)	8 (5.7)	0.046
Insulin	23 (16.3)	21 (15.1)	46 (32.9)	< 0.001

*ACEI* angiotensin-converting enzyme inhibitor, *ARB* angiotensin receptor blocker, *BMI* body mass index, *BP* blood pressure, *CCB* calcium-channel blacker, *eGFR* estimated glomerular filtration rate, *HbA*_*1c*_ glycated hemoglobin A_1c_, *HDL* high-density lipoprotein, *HOMA*-*IR* homeostatic model assessment-insulin resistance, *hsCRP* high-sensitivity C-reactive protein, *LDL* low-density lipoprotein, *LVEF* left ventricular ejection fraction

### Angiographic findings

There were no significant differences in the target vessels, stent counts, stented length, angiographic pre-and post-PCI RD, DS and MLD between the three groups (Table 2). In the overall population, follow-up coronary angiography showed the prevalence of binary angiographic ISR, defined as ≥ 50% DS, was 8.6%. The mean DS was 22.9 ± 16.8%, and the mean net luminal loss and net luminal gain was 0.42 ± 0.88 mm and 1.66 ± 0.83 mm, respectively.

**Table 2:** Lesion and procedural characteristics

Tertiles of CV of HbA_1c_	T1 (0.005–0.045)	T2 (0.045–0.086)	T3 (0.086–0.397)	*P*
Left mainstem lesion	4 (1.82)	4 (1.73)	8 (3.38)	0.363
Left anterior descending lesion	104 (47.27)	96 (41.56)	92 (38.82)	
Circumflex lesion	65 (29.55)	65 (28.14)	71 (29.96)	
Right coronary lesion	47 (21.36)	66 (28.57)	66 (27.85)	
Multivessel disease	109 (77.3)	103 (74.1)	114 (81.4)	0.338
RD, pre-PCI, mm	2.96 ± 0.51	2.91 ± 0.43	2.88 ± 0.44	0.176
%DS pre-PCI	81.82 ± 18.21	78.66 ± 24.75	81.52 ± 24.79	0.278
MLD pre-PCI, mm	0.54 ± 0.55	0.62 ± 0.72	0.55 ± 0.77	0.385
RD, post-PCI, mm	3.17 ± 0.81	3.14 ± 0.94	3.13 ± 0.91	0.858
%DS post-PCI	12.89 ± 13.27	14.69 ± 13.44	13.42 ± 15.06	0.370
MLD post-PCI, mm	2.78 ± 0.87	2.70 ± 0.98	2.73 ± 0.98	0.696
Stent count	1.45 ± 0.64	1.53 ± 0.71	1.48 ± 0.72	0.409
Stented length, mm	36.76 ± 17.88	38.24 ± 20.72	37.11 ± 18.98	0.692

*CV* coefficient of variation, *DS* diameter stenosis, *MLD* minimal luminal diameter, *RD* reference diameter, *PCI* percutaneous coronary intervention

**Fig. 2:**
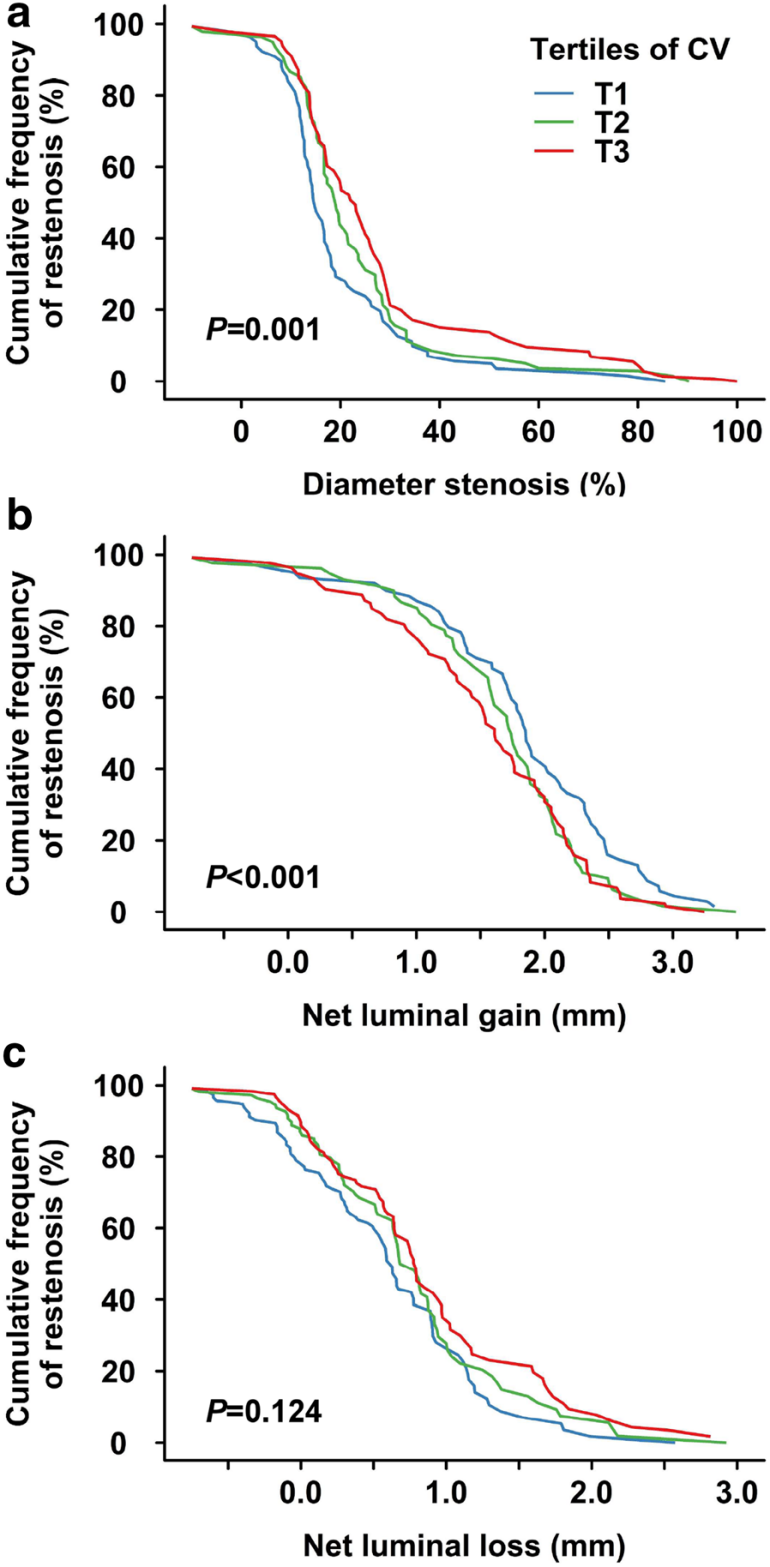
Cumulative frequency of restenosis according to tertiles of CV of HbA_1c_. Cumulative frequency curves for diameter stenosis (**a**), net luminal gain (**b**) and net luminal loss (**c**) at follow-up angiography in subjects with different tertiles of CV of HbA_1c_. CV, coefficient of variation; HbA1c, glycated hemoglobin A_1c_

There was a significant increase in DS across tertiles of CV of HbA_1c_ (Fig. 2a, *P *= 0.001). Compared with subjects with the lowest tertile, a higher percentage of DS was found in the highest tertile (26.63 ± 19.08 vs. 19.29 ± 14.47%, *P *< 0.001). Accordingly, net luminal gain (*P *< 0.001) was step-wisely decreased in subjects with higher HbA_1c_ variability as grouped by all the three measures (Fig. 2b). Although there was no difference in net luminal loss between tertiles of CV (Fig. 2c; *P *= 0.124), it differed significantly between subjects with different tertiles of SD (*P *= 0.023) or VIM (*P *= 0.014) of HbA_1c_ (Additional file 1: Figures S1 and S2). In addition, comparison of HbA_1c_ variability between subjects with and without ISR also showed significantly higher HbA_1c_ variability in ISR patients as analyzed by all the three measures (Additional file 1: Figure S3).

The rate of binary angiographic restenosis was substantially elevated with increasing tertiles of CV of HbA_1c_ (lowest tertile: 5.0%, intermediate tertile: 6.5%, highest tertile: 14.3%; *P *= 0.011). Similar findings were observed when grouping the population based on other measures of HbA_1c_ variability. Meanwhile, increased ISR rate was also observed in patients with higher pre-procedural (baseline HbA_1c_ > 7%: 10.20% vs. HbA_1c_ ≤ 7%: 6.86%, *P *< 0.001) and post-procedural (mean HbA_1c_ > 7%: 11.50% vs. HbA_1c_ ≤ 7%: 4.52%, *P *< 0.001) HbA_1c_ levels.

**Fig. 3:**
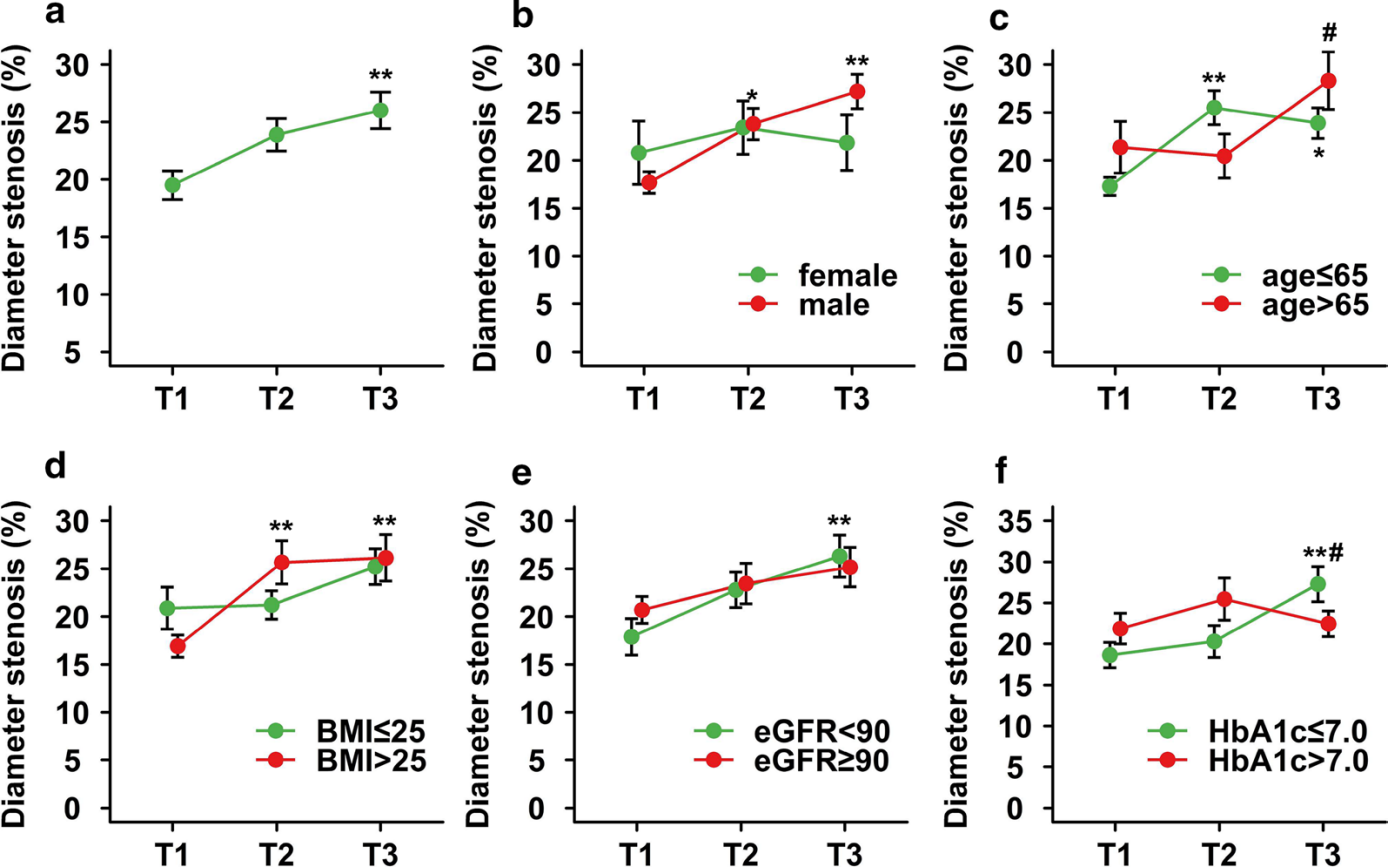
The impact of HbA_1c_ variability on ISR across subgroups. The impact of HbA_1c_ variability on ISR was analyzed in the overall population (**a**) and across subgroups of sex (**b**), age (**c**), dichotomized baseline BMI (**d**), dichotomized baseline eGFR (**e**) and dichotomized baseline HbA_1c_ (**f**). *ISR* in-stent restenosis, *BMI* body mass index, *eGFR* estimated glomerular filtration rate, *HbA1c* glycated hemoglobin A_1c_

The impact of HbA_1c_ variability on ISR was analyzed across subgroups of sex, age, dichotomized baseline BMI, eGFR and HbA_1c_ (Fig. 3). Since the rate of binary ISR was relatively low, DS at follow-up angiography was compared between subgroups. We found DS was increased across tertiles of CV of HbA_1c_ in male but not female subjects. A trend towards higher percentage of DS across the tertiles was more prominent in subjects with higher BMI and poorer renal function, and was similar between two age groups. Interestingly, compared with subjects with higher HbA_1c_ at the time of PCI (HbA_1c_ > 7%), those with lower HbA_1c_ (≤ 7%) appeared to have more severe restenosis when having higher CV of HbA_1c_. There was no significant interaction term between tertiles of CV of HbA_1c_ and these grouping variables, with the solo exception of basal HbA_1c_ level (*P *= 0.010). Dividing subjects by tertiles of SD or VIM yielded similar findings with a little variation (Additional file 1: Figures S4 and S5).

### Multivariate analysis

Multivariate analysis was performed to analyze the association between the incidence of ISR and different measures of HbA_1c_ variability (Table 3). The age- and sex- adjusted HR for ISR in subjects with the highest tertile versus the lowest tertile was 3.26 (95% CI 1.37–7.76). After multivariate adjustment (model 3), the highest tertile conferred a higher risk of ISR as compared to the lowest tertile [2.92 (95% CI 1.18–7.20)]. After additional adjustment for baseline HbA_1c_ (model 4) or the mean HbA_1c_ during follow-up (model 5), the corresponding HR for ISR in the highest tertile versus the lowest tertile remained significant [model 4: 3.28 (95% CI 1.25–8.55); model 5: 3.00 (95% CI 1.14–7.92)]. Similar findings were observed by inclusion of other measures of HbA_1c_ variability into these models. In the full adjustment model (model 5), the highest tertile of SD and VIM were significantly associated with 3.69- and 2.82-fold increased risk (all *P *< 0.05) of ISR compared with the lowest tertile, respectively.

**Table 3:** Multivariate analysis

Tertiles	Model 1		Model 2		Model 3		Model 4		Model 5	
	HR (95% CI)	*P*	HR (95% CI)	*P*	HR (95% CI)	P	HR (95% CI)	*P*	HR (95% CI)	*P*
CV		0.004*		0.008*		0.015*		0.013*		0.023*
T1	Reference	–	Reference	–	Reference	–	Reference	–	Reference	–
T2	1.22 (0.45–3.27)	0.698	1.03 (0.38–2.79)	0.958	0.96 (0.34–2.74)	0.946	1.00 (0.35–2.86)	1.000	0.97 (0.34–2.75)	0.950
T3	3.26 (1.37–7.76)	0.008	3.06 (1.26–7.45)	0.014	2.92 (1.18–7.20)	0.020	3.28 (1.25–8.55)	0.015	3.00 (1.14–7.92)	0.026
SD		0.002*		0.004*		0.008*		0.006*		0.013*
T1	Reference	–	Reference	–	Reference	–	Reference	–	Reference	–
T2	1.60 (0.58–4.40)	0.364	1.27 (0.45–3.59)	0.648	1.11 (0.38–3.20)	0.848	1.21 (0.41–3.55)	0.727	1.15 (0.40–3.37)	0.792
T3	3.88 (1.54–9.77)	0.004	3.53 (1.38–9.06)	0.009	3.28 (1.25–8.65)	0.016	4.02 (1.39–11.61)	0.010	3.69 (1.23–11.05)	0.020
VIM		0.020*		0.042*		0.029*		0.030*		0.032*
T1	Reference	–	Reference	–	Reference	–	Reference	–	Reference	–
T2	1.85 (0.73–4.70)	0.196	1.78 (0.69–4.54)	0.230	1.74 (0.66–4.58)	0.265	1.74 (0.66–4.60)	0.267	1.67 (0.63–4.43)	0.304
T3	2.82 (1.16–6.85)	0.022	2.53 (1.02–6.26)	0.045	2.86 (1.11–7.38)	0.030	2.86 (1.10–7.42)	0.031	2.82 (1.09–7.29)	0.032

Model 1, includes adjustment for age and sex; Model 2: additional adjustment for systolic and diastolic blood pressure, body mass index, non-HDL cholesterol and eGFR; Model 3, additional adjustment for the post-PCI reference diameter of target vessel, total stented length and medication use including oral hypoglycemic agent and insulin; Model 4, model 3 with additional adjustment for baseline HbA_1c_; Model 5, model 3 with additional adjustment for the mean HbA_1c_ during follow-up. * *P* for trend

*CI* confidence interval, *CV* coefficient of variation, *eGFR* estimated glomerular filtration rate, *HbA*_*1c*_ glycated hemoglobin A_1c_, *HDL* high-density lipoprotein, *HR* hazard ratios, *PCI* percutaneous coronary intervention, *SD* standard deviation, *VIM* variability independent of the mean

Inclusion of HbA_1c_ variability led to better risk stratification accuracy. After entering tertiles of CV of HbA_1c_ in the model, 29.4% of subjects with ISR were correctly reclassified to a higher risk category and none was reclassified to a lower category. In patients without ISR, 10.8% were correctly reclassified to a lower risk category and 9.4% were reclassified to a higher category (categories of restenosis: < 10%, 10–20%, ≥ 20%). Accordingly, the categorical NRI was 30.76% [(95% CI 14.78–46.74%), *P *< 0.001], and IDI was 2.81% [(95% CI 0.81–4.82%), *P *= 0.006].

## Discussion

The major findings of the present study are that patients with type 2 diabetes and high post-procedure HbA_1c_ variability tend to have greater neointimal hyperplasia and increased rate of ISR in comparison with those with low HbA_1c_ variability. Evaluation of HbA_1c_ variability by different measures exhibits consistent findings. Consideration of HbA_1c_ variability leads to better risk stratification accuracy of ISR in patients with type 2 diabetes after stent implantation.

### Impact of glycemic level and stability on ISR

Diabetic patients with obstructive and non-obstructive coronary stenosis generally had poor clinical outcomes, owning to diffuse distribution of atherosclerotic lesions, unstable plaques, microvascular dysfunction and higher incidence of in-stent restenosis (ISR) [28, 29]. Compelling evidence has demonstrated a substantially increased rate of ISR in diabetic patients after coronary intervention irrespective of the specific treatment modalities including balloon angioplasty, bare-metal stents (BMS) and DES [7, 8, 30]. However, very few studies analyzed the association of glucose level and stability with the rate of ISR. Corpus et al. found that optimal glucose control (HbA_1c_ ≤ 7%) before catherization was associated with a ~ 2-fold decrease in rate of target vessel revascularization compared to those with suboptimal glucose control (HbA_1c_ > 7%) [10]. A single center prospective study showed that diabetic patients with poor glycemic control at time points both pre- and post-PCI had higher risk of major adverse cardiovascular events (MACE) than non-diabetic patients [14]. In contrast, a retrospective study showed that diabetic patients with good glycemic control (HbA_1c_ ≤ 6.9%) only at the time of PCI, but not at follow-up, was associated with significantly lower incidence of MACE compared to those with poor glycemic control (HbA_1c_ > 6.9%; 18.4% vs. 26.2%, *P *< 0.05) [31]. These studies unanimously suggest that glycemic control at the time of PCI is of importance to prevent subsequent restenosis and adverse cardiovascular outcomes, but with conflicting findings on the effect of post-procedural glycemic control. Actually, glycemic control in these studies was defined according to the cut-off level of HbA_1c_ at certain time points without consideration of glycemic variability. A substantial proportion of patients in these studies received coronary intervention based on BMS, which does not necessarily respond in the same way as that of DES in the process of restenosis under hyperglycemic conditions.

In the present study, all the enrolled patients received DES-based PCI, which reflects the predominant treatment modality in current clinical practice. In accordance with previous reports, we found diabetic patients with poor glycemic control at the time of PCI (HbA_1c_ > 7%) had a 1.49-fold higher rate of ISR than those with good glycemic control (HbA_1c_ ≤ 7%). By grouping patients based on mean HbA_1c_ during follow-up instead, there was an even higher (2.54-fold) increased rate of ISR in subjects with poor versus good glycemic control. Importantly, we for the first time reported that the rate of ISR and angiographic DS were increased across tertiles of HbA_1c_ variability parameters. There was also a trend towards greater net luminal loss and less net luminal gain in patients with higher variability of HbA_1c_. Therefore, previous reports and our findings suggest that both glycemic level and stability are important in the process of ISR after DES implantation in patients with type 2 diabetes. Interestingly, subgroup analysis showed that the impact of HbA_1c_ variability on DS was more prominent in subjects with good (HbA_1c_ ≤ 7%) as compared to those with poor glycemic control (HbA_1c_ > 7%) at the time of PCI. This might be due to the reason that HbA_1c_ reflects both fasting and postprandial glucose levels. In well-controlled diabetic patients (HbA1c < 7.3%), postprandial glucose level is a predominant contributor (70%) to HbA_1c_ and this contribution decreases progressively with increasing level of HbA_1c_ [32]. Hence, variability of postprandial glucose might be more important than fasting glucose in the development of ISR and this hypothesis awaits further investigation.

Currently, there is no universally accepted “gold standard” to quantify glycemic variability. In this study, we assessed HbA_1c_ variability by three different measures. In addition to SD, CV and VIM were employed to adjust for mean HbA_1c_ during follow-up. VIM was calculated based on logarithmic curve fitting to eliminate its correlation with mean HbA_1c_, and CV is relatively simple and more feasible in clinical practice. Analysis of HbA_1c_ variability by all of these three measures yielded similar findings. After adjusting for mean HbA_1c_ level during follow-up, different measures of HbA_1c_ variability remained significantly associated with the incidence of ISR. Inclusion of HbA_1c_ variability led to significantly increased risk prediction accuracy compared to the model that only included conventional risk factors, lesion and procedure characteristics, and mean HbA_1c_. These findings support the notion that HbA_1c_ variability is independent of glycemic level in association with ISR. Actually, previous secondary analyses of data from DCCT [19] and Finnish Diabetic Nephropathy (FinnDiane) Study [33] revealed that HbA_1c_ variability is an independent predictor of incident microalbuminuria, progression of renal disease and also incident cardiovascular events in patients with type 1 diabetes. A study analyzing 58,832 patients with type 2 diabetes in a large primary care database in England showed that HbA_1c_ variability was strongly associated with overall mortality and emergency hospitalization and not explained by mean HbA_1c_ [34]. A single center prospective study found that elevated admission glycemic variability appears even more important than admission glucose in predicting 1-year MACE in patients with acute myocardial infarction [35]. Therefore, although it is hard to tease out the relative effect of HbA_1c_ variability after controlling for HbA_1c_ level in the process of ISR, HbA_1c_ variability appears to function independently in various diabetic complications including ISR.

### Possible mechanisms

It is unclear the specific mechanism by which HbA_1c_ variability affects the development of restenosis in diabetic patients. Based on previous clinical and basic science studies, potential mechanisms include: First, hyperglycemia and glycemic fluctuation directly and indirectly stimulate the production of reactive oxygen species, inflammatory and metabolic cytokines, which are essential players in the development of adverse myocardial and vascular remodeling, and worse clinical outcomes both in patients with or without diagnosed diabetes [36–40]. Second, glycemic variability is strongly correlated with postprandial β-cell dysfunction in type 2 diabetic patients using OHA. Consistently, we found postprandial insulin level was lower and insulin resistance was higher in patients with the highest tertile of CV than those with the lowest tertile [41]. Given that insulin resistance is an established contributory factor in restenosis, the impact of HbA_1c_ variability on ISR may also be secondary to insulin resistance. Third, dysregulated glucose homeostasis is associated with endothelial dysfunction and higher risk of cardiovascular events [42, 43]. Mounting evidence suggests that endothelial dysfunction is an important predictor of restenosis after stent implantation [44, 45]. Hence, endothelial dysfunction and delayed reendothelialization may serve as an important underlying mechanism in the development of ISR in conditions of high glycemic variability.

### Study limitation

Our findings should be interpreted in the context of following limitations. First, this study is a retrospective analysis based on prospectively collected data, and all the enrolled patients were from a single center. Second, fluctuations in fasting plasma glucose (FPG) and HbA_1c_ appear to function differentially in the process of diabetic complications [19, 46]. Variability of FPG was not analyzed in this study, which may have different features or function in different phases as compared to that of HbA_1c_. Moreover, conditions that affect erythrocyte turnover may also affect HbA_1c_ level. Third, coronary lesions and restenosis were analyzed by QCA. Intravascular imaging techniques such as intravascular ultrasound would provide more accurate assessments. Fourth, this study was not designed to analyze the predictive value of HbA_1c_ variability for hard endpoints in diabetic patients underwent PCI. Although we found ISR rate was significantly elevated in patients with high variability of HbA_1c_, whether these patients suffer higher risk of cardiovascular mortality remains inconclusive.

## Conclusions

In conclusion, our findings suggest that greater visit-to-visit HbA_1c_ variability is associated with higher incidence of ISR in patients with type 2 diabetes after stent implantation. Variability of HbA_1c_ adds to mean level for risk prediction of ISR. Measures targeting both glycemic level and stability may provide favorable effects to reduce the incidence of ISR and improve clinical outcomes in patients with type 2 diabetes after PCI.

## Supplementary information

**Additional file 1:** **Figure S1** Cumulative frequency curves for diameter stenosis (DS, A), net luminal gain (B) and net luminal loss (C) at follow-up angiography in subjects with different tertiles of SD of HbA_1c_. **Figure S2** Cumulative frequency curves for diameter stenosis (DS, A), net luminal gain (B) and net luminal loss (C) at follow-up angiography in subjects with different tertiles of VIM of HbA_1c_. **Figure S3** Comparison of different measures of intraindividual variability of HbA1c between patients with and without ISR. **Figure S4** Subgroup analysis of diameter stenosis (DS) at follow-up angiography based on tertiles of SD of HbA_1c_. Data are expressed as mean ± confidence interval (CI). **T1** the lowest tertile; **T2** intermediate tertile; **T3** the highest tertile. *P < 0.05, **P < 0.01 vs. the lowest tertile; #*P* < 0.05 vs. intermediate tertile. **Figure S5** Subgroup analysis of diameter stenosis (DS) at follow-up angiography based on tertiles of VIM of HbA_1c_. Data are expressed as mean ± confidence interval (CI). **T1** the lowest tertile; **T2** intermediate tertile; **T3** the highest tertile. *P < 0.05, **P < 0.01 vs. the lowest tertile; #*P* < 0.05 vs. intermediate tertile.

## Data Availability

The datasets used and/or analyzed during the current study are available from the corresponding author on reasonable request.

## Abbreviations

BMI: Body mass index
BMS: Bare-metal stents
BSA: Body surface area
CAD: Coronary artery disease
CI: Confidence interval
CV: Coefficient of variation
DES: Drug-eluting stent(s)
DS: Diameter stenosis
eGFR: Estimated glomerular filtration rate
FPG: Fasting plasma glucose
HDL-C: High-density lipoprotein cholesterol
HR: Hazard ratios
hs-CRP: High sensitive C-reactive protein
IDI: Integrated discrimination improvements
ISR: In-stent restenosis
LDL-C: Low-density lipoprotein cholesterol
MACE: Major adverse cardiovascular events
MLD: Minimal lumen diameter
NRI: Net reclassification improvements
NT-proBNP: N-terminal pro-B-type natriuretic peptide
OHA: Oral hypoglycemic agent
PCI: Percutaneous coronary intervention
QCA: Quantitative coronary analysis
RD: Reference diameter
SD: Standard deviation
VIM: Variability independent of the mean
