# Supplementary material for: Visit-to-visit HbA1c variability is associated with in-stent restenosis in patients with type 2 diabetes after percutaneous coronary intervention
Source: Cardiovasc Diabetol. 2020 Sep 4;19:133. doi: 10.1186/s12933-020-01111-7 (PMC7472579; doi:10.1186/s12933-020-01111-7)
Supplement: Supplementary file 1 — Additional file 1: Figure S1 Cumulative frequency curves for diameter stenosis (DS, A), net luminal gain (B) and net luminal loss (C) at follow-up angiography in subjects with different tertiles of SD of HbA1c. Figure S2 Cumulative frequency curves for diameter stenosis (DS, A), net luminal gain (B) and net luminal loss (C) at follow-up angiography in subjects with different tertiles of VIM of HbA1c. Figure S3 Comparison of different measures of intraindividual variability of HbA1c between patients with and without ISR. Figure S4 Subgroup analysis of diameter stenosis (DS) at follow-up angiography based on tertiles of SD of HbA1c. Data are expressed as mean ± confidence interval (CI). T1 the lowest tertile; T2 intermediate tertile; T3 the highest tertile. *P < 0.05, **P < 0.01 vs. the lowest tertile; #P < 0.05 vs. intermediate tertile. Figure S5 Subgroup analysis of diameter stenosis (DS) at follow-up angiography based on tertiles of VIM of HbA1c. Data are expressed as mean ± confidence interval (CI). T1 the lowest tertile; T2 intermediate tertile; T3 the highest tertile. *P < 0.05, **P < 0.01 vs. the lowest tertile; #P < 0.05 vs. intermediate tertile. [file 12933_2020_1111_MOESM1_ESM.pdf]

SUPPLEMENTAL MATERIAL

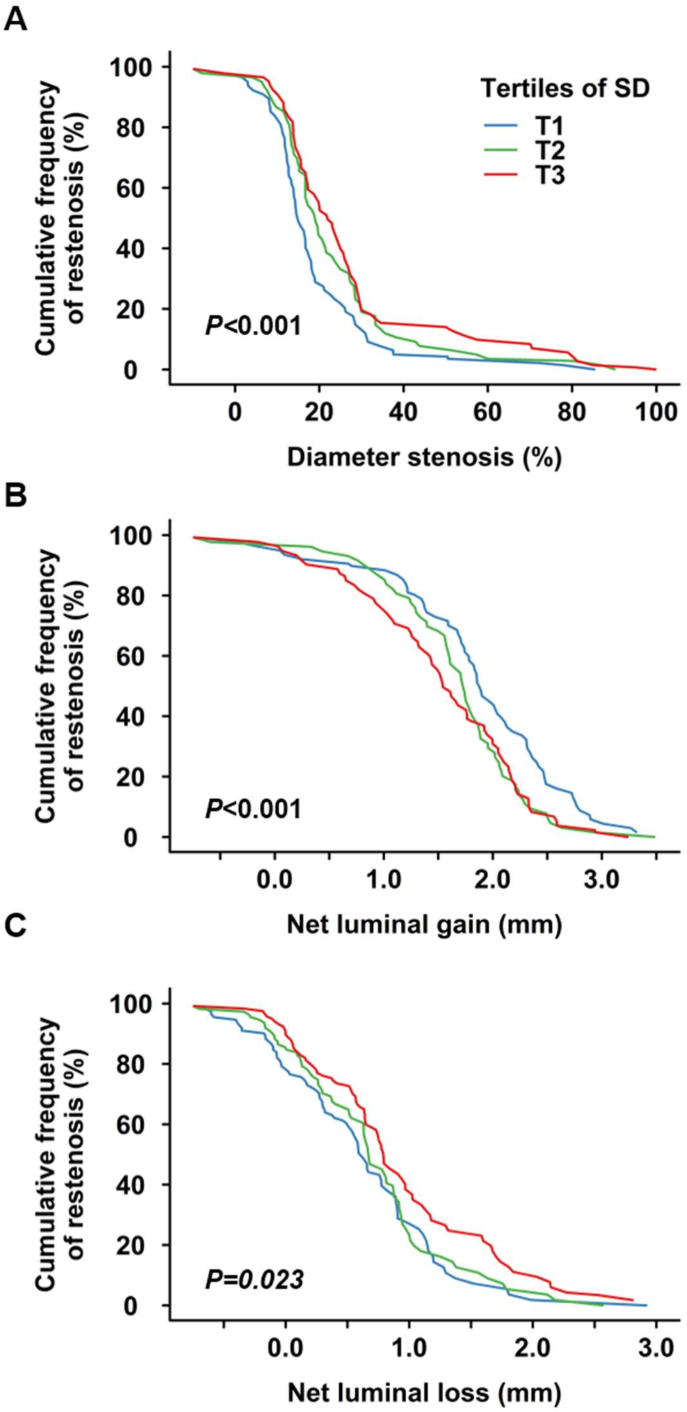

Supplementary figure I. Cumulative frequency curves for diameter stenosis (DS, A), net luminal gain (B) and net luminal loss (C) at follow-up angiography in subjects with different tertiles of SD of HbA_{1c}.

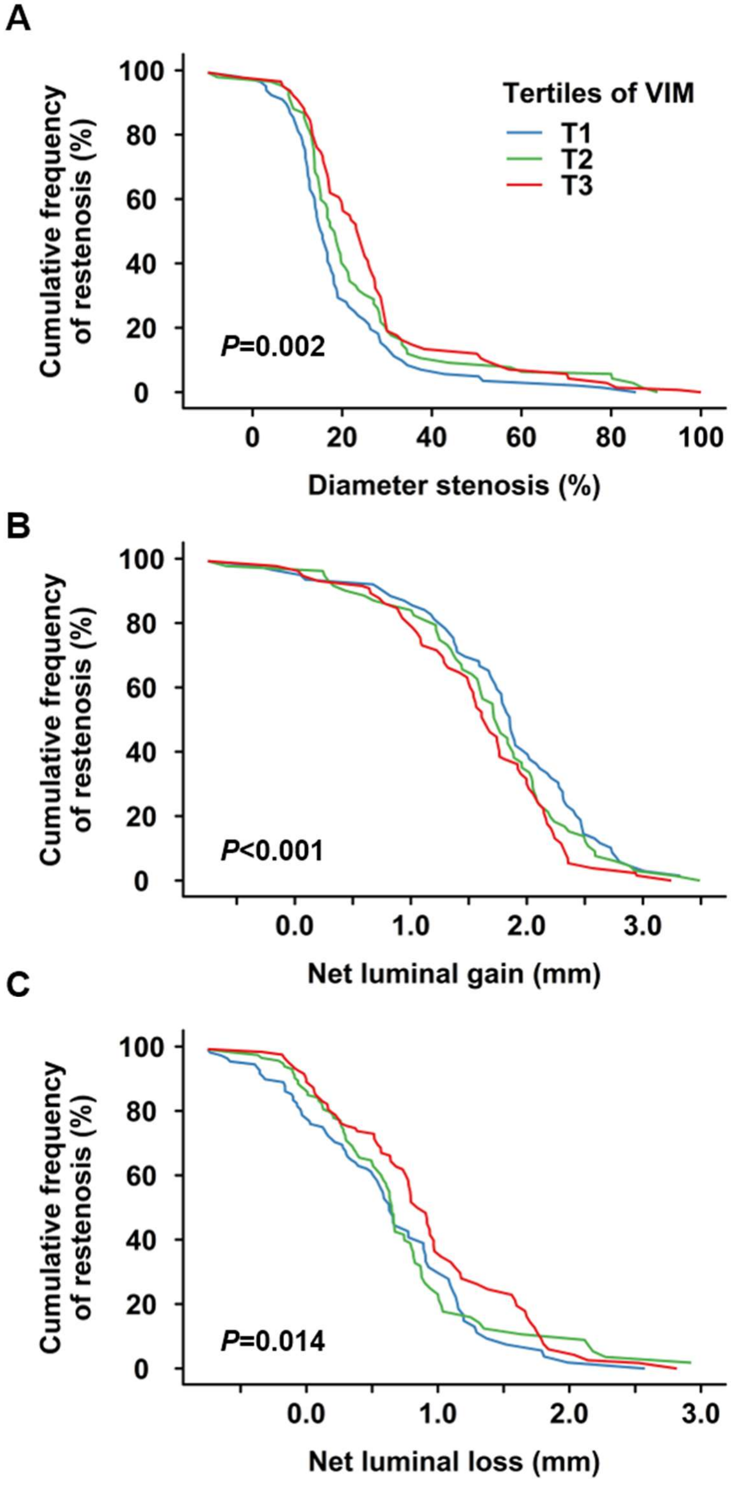

Supplementary figure II. Cumulative frequency curves for diameter stenosis (DS, A), net luminal gain (B) and net luminal loss (C) at follow-up angiography in subjects with different tertiles of VIM of HbA_{1c}.

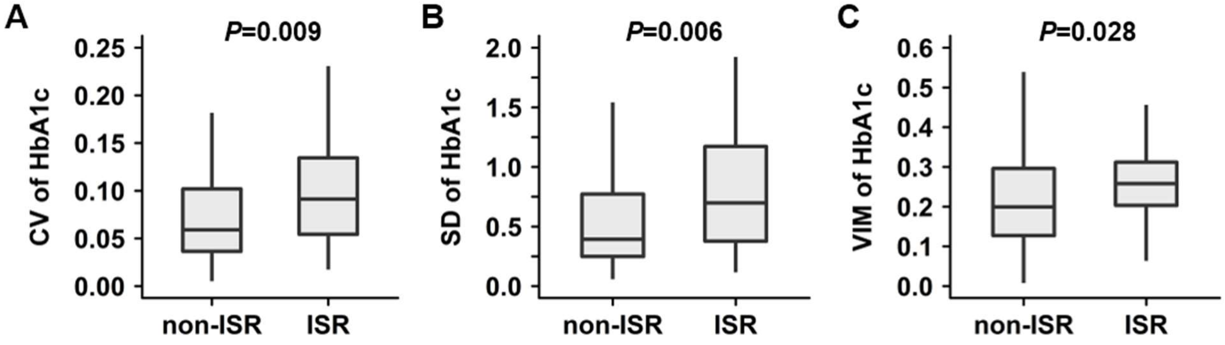

Supplementary figure III. Comparison of different measures of intraindividual variability of HbA_{1c} between patients with and without ISR.

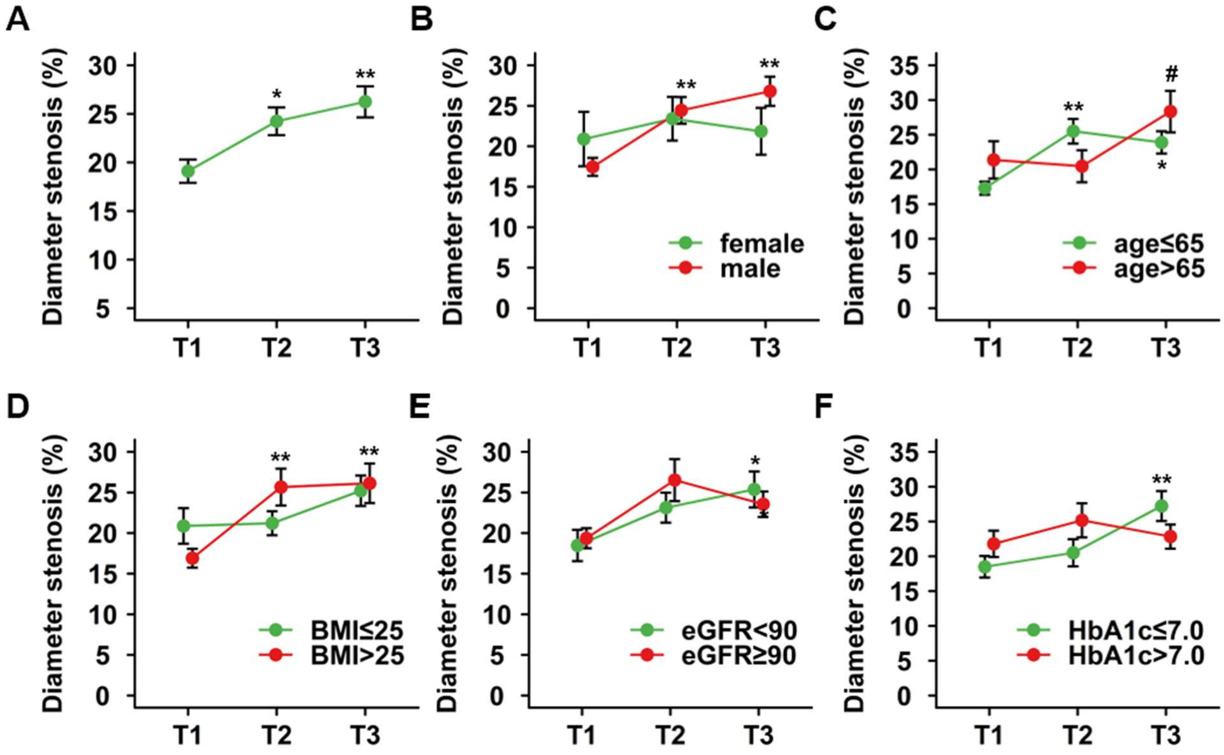

Supplementary figure IV. Subgroup analysis of diameter stenosis (DS) at follow-up angiography based on tertiles of SD of HbA_{1c}. Data are expressed as mean ± confidence interval (CI). T1, the lowest tertile; T2, intermediate tertile; T3, the highest tertile. **P*<0.05, ***P*<0.01 vs. the lowest tertile; #*P*<0.05 vs. intermediate tertile.

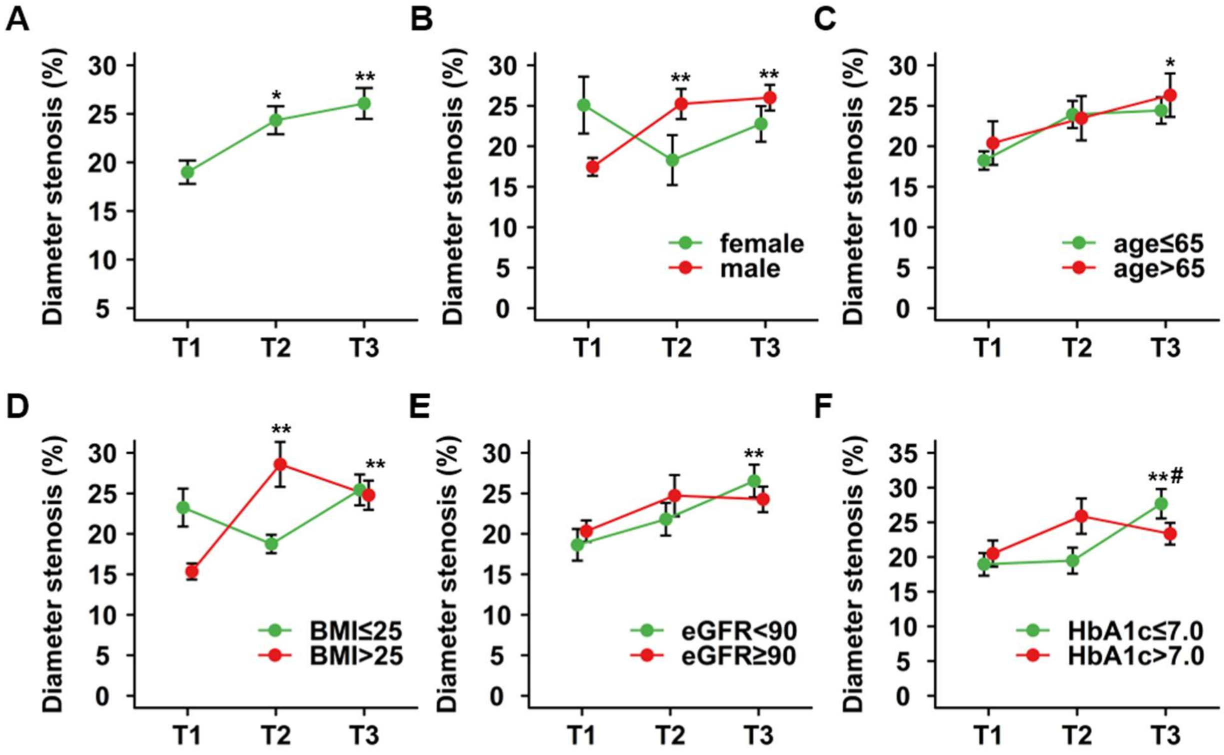

Supplementary figure V. Subgroup analysis of diameter stenosis (DS) at follow-up angiography based on tertiles of VIM of HbA_{1c}. Data are expressed as mean ± confidence interval (CI). T1, the lowest tertile; T2, intermediate tertile; T3, the highest tertile. **P*<0.05, ***P*<0.01 vs. the lowest tertile; #*P*<0.05 vs. intermediate tertile.
